# Distribution of health literacy strengths and weaknesses across socio-demographic groups: a cross-sectional survey using the Health Literacy Questionnaire (HLQ)

**DOI:** 10.1186/s12889-015-2056-z

**Published:** 2015-07-21

**Authors:** Alison Beauchamp, Rachelle Buchbinder, Sarity Dodson, Roy W. Batterham, Gerald R. Elsworth, Crystal McPhee, Louise Sparkes, Melanie Hawkins, Richard H. Osborne

**Affiliations:** Public Health Innovation, Population Health Strategic Research Centre, School of Health and Social Development, Deakin University, 221 Burwood Highway, Melbourne, VIC 3125 Australia; Department of Epidemiology and Preventive Medicine, School of Public Health and Preventive Medicine, Monash University, Melbourne, Australia; Monash Department of Clinical Epidemiology, Cabrini Hospital, Malvern, VIC Australia; School of Nursing, Monash University, Melbourne, Australia

**Keywords:** Health literacy, Health inequities, Chronic disease, Culturally and linguistically diverse, Health Literacy Questionnaire, HLQ

## Abstract

**Background:**

Recent advances in the measurement of health literacy allow description of a broad range of personal and social dimensions of the concept. Identifying differences in patterns of health literacy between population sub-groups will increase understanding of how health literacy contributes to health inequities and inform intervention development. The aim of this study was to use a multi-dimensional measurement tool to describe the health literacy of adults in urban and rural Victoria, Australia.

**Methods:**

Data were collected from clients (n = 813) of 8 health and community care organisations, using the Health Literacy Questionnaire (HLQ). Demographic and health service data were also collected. Data were analysed using descriptive statistics. Effect sizes (ES) for standardised differences in means were used to describe the magnitude of difference between demographic sub-groups.

**Results:**

Mean age of respondents was 72.1 (range 19–99) years. Females comprised 63 % of the sample, 48 % had not completed secondary education, and 96 % reported at least one existing health condition. Small to large ES were seen for mean differences in HLQ scales between most demographic groups. Compared with participants who spoke English at home, those not speaking English at home had much lower scores for most HLQ scales including the scales ‘Understanding health information well enough to know what to do’ (ES −1.09 [95 % confidence interval (CI) -1.33 to −0.84]), ‘Ability to actively engage with healthcare providers’ (ES −1.00 [95 % CI −1.24, −0.75]), and ‘Navigating the healthcare system’ (ES −0.72 [95 % CI −0.97, −0.48]). Similar patterns and ES were seen for participants born overseas compared with those born in Australia. Smaller ES were seen for sex, age group, private health insurance status, number of chronic conditions, and living alone.

**Conclusions:**

This study has revealed some large health literacy differences across nine domains of health literacy in adults using health services in Victoria. These findings provide insights into the relationship between health literacy and socioeconomic position in vulnerable groups and, given the focus of the HLQ, provide guidance for the development of equitable interventions.

## Background

Health literacy refers to the characteristics and social resources needed for people to access, understand and use information to make decisions about health. Health literacy includes the capacity to communicate, assert and enact these decisions [1–3]. Individuals and communities can have health literacy strengths as well as limitations that influence how effectively they engage with health information and services. Health services can also have strengths and limitations in the way in which they respond to people with different health literacy requirements. We have adopted the term ‘health literacy responsiveness’ to describe the ways in which health organisations and products (e.g. signage and medicine labels) enable health information and services to be accessible to people with different health literacy abilities [3]. For a service to be ‘health literacy responsive’, it not only needs to implement practices that support all people regardless of their levels of health literacy [3, 4] but also needs to understand the health literacy strengths and limitations of people in the communities that it seeks to serve.

The importance of health literacy for public health and as a determinant of health equity is becoming increasingly evident. Lower health literacy scores, measured as reading ability and numeracy, have been shown in several studies to be associated with higher avoidable hospitalisation rates [5], decreased ability to self-care [6], poorer health outcomes, and higher mortality rates [7–9]. Surveys in Australia, Canada, New Zealand, and the USA have suggested that over half the population may have substantial difficulties with reading and numeracy associated with health-related information and tasks [10–14]. Similar surveys have also found that health literacy independently predicts health outcomes [15, 16] even after controlling for socioeconomic position (SEP) [17]. However, these surveys have not provided substantive insight into the causes and implications of sub-optimal health literacy, other than indicating a relationship between general education and literacy.

Given an increasing recognition of the importance of health literacy, it is useful to consider the concept in relation to priority health issues. Globally, chronic disease has a rapidly increasing prevalence due to increases in lifespan, and changes to lifestyle [18, 19]. Notably, the burden of chronic disease is greatest among those who are more socially and economically disadvantaged, contributing to significant inequalities in mortality and morbidity [20]. The impact of low health literacy in people with chronic disease is considerable. Optimal chronic care involves patients frequently accessing the healthcare system and actively self-managing their health condition [21]. The demands upon people in relation to functional, social, and cognitive skills are substantial, as are their information and support requirements [18]. People who have limited health literacy engage less effectively in disease management activities, resulting in poorer outcomes and increased burden [22, 23]. Interventions and resources to support people with chronic disease and optimise access to services must be appropriate to their health literacy needs if they are to be effective [24, 25]. An accurate and complete understanding of the health literacy needs of this group is therefore required.

Until recently, the tools available to measure health literacy have predominantly focused on numeracy skills, reading ability or language comprehension [26–28], and have sought to produce a single summative health literacy score. Other aspects of health literacy that are explicit in most definitions of the concept have been largely overlooked [29, 30]. Tools have, for example, omitted measurement of ability to engage with healthcare providers, navigate the health system, or critique health information [11, 29]. Incomplete capture of key elements of a multi-dimensional concept such as health literacy results in ‘construct under-representation’ [31] which compromises the validity of study conclusions. Further, the presentation of health literacy as a single construct misrepresents its complexity and, importantly, restricts public health professionals, clinicians and policy makers in their ability to identify how best to intervene to improve health literacy-related outcomes. In response to this, the Health Literacy Questionnaire (HLQ) [30] was developed using an extensive grounded approach that explicitly sought to represent a holistic picture of an individual’s health literacy.

This study aims to describe the health literacy of a sample of adults who access local government, community health and acute healthcare services in urban and rural Victoria, Australia and who are participating in a larger study called the Ophelia (Optimising Health Literacy and Access in Victoria) Study [32]. Health literacy is assessed using the HLQ to measure people’s strengths and limitations in being able to access, understand and use health information and health services. Results will provide a detailed picture of the health literacy profile of the study population.

## Methods

### Study design

This paper reports the first phase of the Ophelia Study: a collaborative project that aims to build a Health Literacy Response Framework, including developing and testing a suite of health literacy interventions. The full protocol of Ophelia is described elsewhere [32]. For the first phase, a cross-sectional survey was undertaken using the HLQ to describe the health literacy profile of people using community-based health services across urban and rural Victoria. This paper describes findings from this survey.

### Setting

Similar to the rest of Australia, Victoria (population 5.8 million) [33] has a universal healthcare system that operates alongside optional private health insurance. We selected four of the eight regions of Victoria [34] to represent a diverse range of socioeconomic and geographical characteristics of the state. Within each of the four selected regions, organisations providing Home and Community Care (HACC) services, Hospital Admission Risk Programs (HARP) or community nursing and other chronic disease services were invited to participate through an expression of interest process. Eight organisations were selected to participate. Criteria for inclusion are described in detail elsewhere [32]. Selected organisations included two local governments, two HARP programs (one included clients from a community health service), three community health centres, and one home-based nursing service. Of these eight organisations, five were based in metropolitan urban areas, one in an outer metropolitan area, and two in rural areas. There was variability in the acute, chronic and support services offered by the organisations, ranging from episodic allied health services to more comprehensive programs such as chronic and complex care or aged care packages.

### Participants

The sample for this study comprised people attending one of the eight participating organisations. Each organisation selected a target group of clients based on a service-provision priority. Given the nature of services provided, the majority of participants were expected to have a chronic health condition although this was not a pre-requisite for inclusion. For example, one organisation chose to focus on understanding the health literacy of clients with diabetes, while another focused on clients who were socially isolated. Staff from each organisation collected data from a representative sample of clients within their target group, using consecutive methods of recruitment where feasible. Staff were provided with training and ongoing support for maximising recruitment of typical clients who attended their service. Strategies for recruiting clients who are traditionally ‘harder to reach’ included offering the option of verbal consent, reading the questionnaire aloud, or giving people the option of taking the questionnaire home to complete with family or friends. Participant selection criteria were deliberately unrestrictive, but required that participants should be cognitively able to provide informed consent to participate, and be over the age of 18 years. Potential participants were invited to take part either by their attending clinician, by other healthcare workers delivering direct services, or, in one case through postal survey.

### Ethics

Human Research Ethics Committee (HREC) approval was obtained from Deakin and Monash Universities as well as the HREC committees of each organisation as required. Informed written consent was obtained from each participant. To maximise participation of people who may have low literacy, an oral consent option was also offered where a witness, who was not involved in the respondent’s direct care, signed on behalf of the respondent.

### Data collection

Data collection took place between July 2013 and February 2014. Participants were provided with the HLQ to self-complete, or a healthcare worker, research assistant, or family member verbally administered the questionnaire. The HLQ and patient information and consent form were translated into several common community languages (Chinese, Greek, Italian, and Vietnamese) for non-English-speaking participants. Self-reported demographic and health data were also collected: age, sex, living alone or with others, private health insurance status, indigenous status, country of birth (Australia or overseas), whether English was the main language spoken at home, and pre-existing health conditions. Educational attainment was coded as ‘did not complete secondary education’ or ‘completed secondary education’, with this variable derived from a 5-category scale that ranged from primary school or less to university educated. Participants were also asked if they were assisted to answer the questionnaire.

The HLQ is a 44 item measure that captures the concept of health literacy across nine distinct domains (measured using one scale per domain). The nine scales are:

1. Feeling understood and supported by healthcare providers;
2. Having sufficient information to manage my health;
3. Actively managing my health;
4. Social support for health;
5. Appraisal of health information;
6. Ability to actively engage with healthcare providers
7. Navigating the healthcare system;
8. Ability to find good health information;
9. Understand health information enough to know what to do) [30].

Each of the nine scales contains between 4 and 6 items that are scored as a graded response. There are four response options for items in the first 5 scales: strongly disagree, disagree, agree and strongly agree. Scales 6–9 have a range of five possible responses: cannot do, very difficult, quite difficult, easy, and very easy. Scale scores were devised by summing the item scores and dividing by the number of items in the scale. Scale scores range between 1 and 4 for the first 5 scales, and 1 and 5 for scales 6 to 9.

Each of the nine scales has been found to be highly reliable (composite reliability range from 0.8 to 0.9 for each of the 4- to 6-item scales) [30]. One-factor confirmatory factor analysis models using Bayesian structural equation modelling (Bayesian SEM [35]) confirmed the homogeneity of all scales [36]. Given that in this paper, and in future studies, comparison of HLQ scale scores across disparate groups of individuals (i.e., different settings, age groups, sex, and cultural background) will be undertaken, we established that the scales were, in the great majority of categories, measurement invariant [36]. We thus established that comparisons between groups based on summed or averaged scale scores have little or no bias. Measurement invariance was not fully established for scales 7–9 for age, education level and language spoken [36].

### Statistical analysis

HLQ scale scores and demographic data were analysed using SPSS Version 21 [37]. The expectation maximization (EM) algorithm was used to impute missing HLQ item scores where there were fewer than 2 missing values from scales with 4 to 5 items and fewer than 3 missing values from the scale with 6 items. Demographic data between sites were compared using the chi-square test.

For all HLQ scales, while responses covered the full range of the scales with modest or no floor or ceiling effects, assumptions of normal distribution were not met. Scales 1 and 6–9 also violated homogeneity of variances. We therefore used robust analysis of variance (ANOVA) for analysis of HLQ scores using the Welch method [38]. Where required, post hoc testing was undertaken using the Games-Howell method of multiple mean comparisons.

Effect sizes (ES) for standardised differences in means between demographic groups were calculated in Stata 13 [39], using Cohen’s d (calculated as the difference between the two means, divided by the pooled standard deviation (SD) of both means) [40] with interpretation of ES as follows: ‘small’ ES >0.20-0.50, ‘medium’ ES approximately 0.50-0.80, and ‘large’ ES >0.80 [40]. This was not a population-based study and no *a priori* sample size calculation was undertaken. Where relevant, 95 % confidence intervals were calculated. A p-value of <0.05 was assumed for statistical significance.

## Results

A total of 813 participants were recruited from across eight organisations, ranging from 72 to 132 participants per site. Demographic characteristics of the sample are shown in Table 1.

**Table 1:** Demographic data for overall sample n = 813

	n	(%)	Missing data (n)
Female	505	(62.9)	10
Age ≥65 yrs	607	(77.0)	25
Lives alone	337	(43.3)	35
Lower education	376	(48.0)	30
Born in Australia	541	(67.2)	8
English spoken at home	723	(90.8)	17
Identifies as Indigenous/Torres Strait Islander	11	(1.4)	16
Arthritis	399	(50.3)	19
Back Pain	338	(42.5)	18
Heart disease	325	(41.0)	20
Lung disease	176	(22.2)	19
Cancer	77	(9.7)	19
Depression/Anxiety	238	(29.9)	18
Diabetes Mellitus	300	(37.7)	18
Stroke	82	(10.3)	18
≥4 chronic conditions	276	(34.0)	23
Reports no health condition	35	(4.4)	18
Private Health Insurance	298	(37.5)	19
Lives in rural area	169	(20.8)	0
Assistance with questionnaire	291	(36.6)	18

The mean (SD) age of participants was 72.1 years (14.5), range 19 to 99, with 23.0 % of participants aged under 65 years (8.0 % were aged under 50 years). This was broadly the expected age of this sample, given that 34.0 % had at least 4 chronic conditions. Females comprised 62.9 % of the sample, 67.2 % were born in Australia, and 90.8 % spoke English as their main language at home. Approximately 37 % of the sample required assistance to complete the questionnaire.

**Fig 1:**
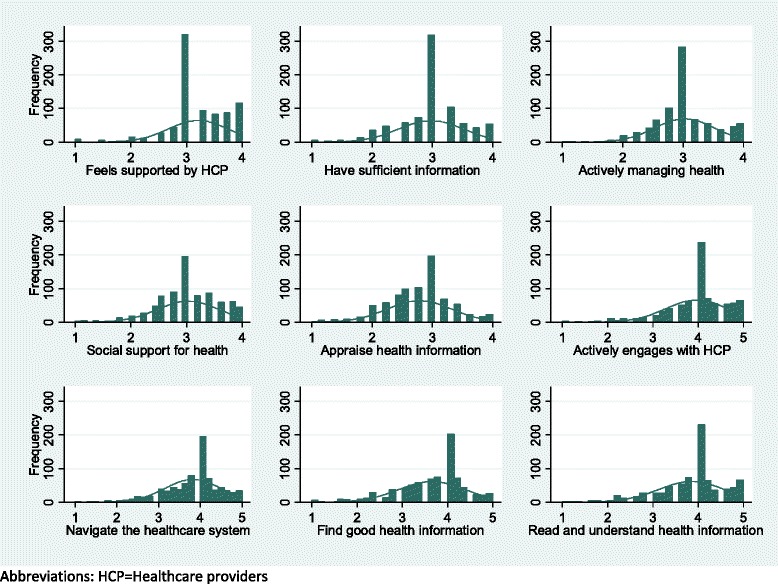
Distribution of HLQ scales for the overall sample

Mean scores for each HLQ scale are shown in Table 2, with the distribution of each scale shown in Fig. 1. For the first 5 scales, answered using response options ranging from strongly disagree to strongly agree (range 1 to 4), the highest overall score was seen for the scale ‘Feeling understood and supported by healthcare providers’ (mean score 3.21 (SD 0.54)). The lowest score was for ‘Appraisal of health information’ (mean score 2.78 (SD 0.54)). For the last 4 scales (range 1 to 5: cannot do to very easy), highest and lowest scores were for ‘Ability to engage with healthcare providers’ (mean score 3.97 (SD 0.69)) and ‘Ability to find good health information’ (mean score 3.65 (SD 0.75)), respectively. A total of 23 participants were shown to be missing data across one or more HLQ scales: no statistically significant differences were seen between those missing and not missing HLQ data.

**Table 2:** Health Literacy Questionnaire (HLQ) scores for overall sample

	Mean (SD) [95 % CI]	Missing data (n)
HLQ scale		
	Range 1 (lowest) -4 (highest)	
1. Feeling understood and supported by healthcare professionals	3.21 (0.54) [3.17, 3.25]	2
2. Having sufficient information to manage my health	2.98 (0.54) [2.94, 3.01]	3
3. Actively managing my health	3.02 (0.50) [2.99, 3.06]	4
4. Social support for health	3.03 (0.55) [2.99, 3.07]	3
5. Appraisal of health information	2.78 (0.54) [2.75, 2.82]	7
	Range 1 (lowest) -5 (highest)	
6. Ability to actively engage with healthcare professionals	3.97 (0.69) [3.92, 4.02]	15
7. Navigating the healthcare system	3.82 (0.67) [3.78, 3.87]	12
8. Ability to find good health information	3.65 (0.75) [3.60, 3.71]	15
9. Understand health information enough to know what to do	3.85 (0.74) [3.81, 3.91]	13

Abbreviations = *SD* Standard deviation, *CI* confidence interval

Table 3 shows patterns of HLQ scores according to demographic status. Note that the following description of demographic differences across the HLQ scales, while focused on effect sizes (ES), includes all those differences found to be statistically significant at probability of <0.05. The ES of several of these differences is small (i.e., <0.2).

**Table 3:** Association between HLQ scores and demographic characteristics

		Feel understood & supported by health care professionals	Have sufficient information to manage health	Actively managing my health	Social support for health	Appraisal of health information	Ability to actively engage with health care professionals	Navigating the Healthcare System	Ability to find good health information	Understand health information
		Mean score (SD)								
Sex	Male	3.26 (0.54)	2.98 (0.55)	2.99 (0.50)	**3.10 (0.55)**	2.74 (0.56)	**4.04 (0.59)**	3.85 (0.63)	3.67 (0.67)	3.81 (0.69)
		n = 296	n = 296	n = 295	n = 296	n = 294	n = 293	n = 294	n = 292	n = 294
	Female	3.19 (0.53)	2.97 (0.54)	3.05 (0.49)	**2.99 (0.55)**	2.81 (0 53)	**3.93 (0.74)**	3.82 (0.70)	3.65 (0.79)	3.89 (0.77)
		n = 505	n = 505	n = 504	n = 504	n = 502	n = 498	n = 500	n = 499	n = 499
Effect size for sex (95 % CI)		0.12 (−0.02, 0.27)	0.02 (−0.12, 0.16)	−0.11 (−0.26, 0.03)	0.19 (0.05, 0.33)	−0.14 (−0.28, 0.01)	0.16 (0.02, 0.30)	0.05 (−0.09, 0.19)	0.02 (−0.12, 0.16)	−0.11 (−0.25, 0.03)
Age group	<65 yr	3.23 (0.57)	2.93 (0.59)	**2.95 (0.50)**	**2.94 (0.58)**	2.80 (0.55)	3.92 (0.73)	3.74 (0.71)	3.65 (0.73)	3.86 (0.74)
		n = 181	n = 181	n = 181	n = 181	n = 181	n = 179	n = 179	n = 179	n = 179
	≥65 yr	3.21 (0.53)	2.99 (0.53)	**3.05 (0.50)**	**3.06 (0.55)**	2.77 (0.54)	4.00 (0.67)	3.86 (0.66)	3.66 (0.75)	3.87 (0.73)
		n = 605	n = 604	n = 604	n = 604	n = 600	n = 596	n = 599	n = 596	n = 598
Effect size age (95 % CI)		−0.02 (−0.19, 0.15)	0.12 (−0.04, 0.29)	0.20 (0.03, 0.37)	0.22 (0.06, 0.39)	−0.05 (−0.22, 0.11)	0.12 (−0.05, 0.28)	0.18 (0.01, 0.34)	0.01 (−0.15, 0.18)	0.02 (−0.14, 0.19)
Completed secondary education	No	3.19 (0.53)	2.97 (0.54)	2.99 (0.51)	3.02 (0.54)	**2.71 (0.53)**	3.95 (0.68)	3.81 (0.67)	**3.56 (0.79)**	**3.75 (0.78)**
		n = 375	n = 375	n = 374	n = 375	n = 374	n = 371	n = 373	n = 372	n = 373
	Yes	3.23 (0.55)	2.99 (0.55)	3.05 (0.49)	3.04 (0.57)	**2.84 (0.54)**	3.99 (0.70)	3.84 (0.68)	**3.74 (0.69)**	**3.95 (0.69)**
		n = 436	n = 435	n = 435	n = 435	n = 432	n = 427	n = 428	n = 426	n = 427
Effect size education (95 % CI)		0.09 (−0.05, 0.22)	0.04 (−0.10, 0.18)	0.13 (−0.01, 0.26)	0.05 (−0.09, 0.19)	0.25 (0.11, 0.39)	0.06 (−0.08, 0.20)	0.04 (−0.10, 0.18)	0.25 (0.11, 0.39)	0.28 (0.14, 0.41)
Health insurance	No	3.21 (0.51)	2.98 (0.53)	**2.98 (0.49)**	**3.00 (0.55)**	2.78 (0.54)	3.95 (0.71)	3.80 (0.71)	3.62 (0.79)	**3.79 (0.79)**
		n = 495	n = 495	n = 493	n = 494	n = 491	n = 487	n = 487	n = 485	n = 487
	Yes	3.23 (0.58)	2.98 (0.57)	**3.10 (0.51)**	**3.08 (0.56)**	2.79 (0.53)	4.00 (0.66)	3.88 (0.61)	3.72 (0.66)	**3.99 (0.64)**
		n = 297	n = 297	n = 297	n = 297	n = 296	n = 293	n = 296	n = 295	n = 295
Effect size insurance (95 %, CI)		−0.03 (−0.18, 0.11)	−0.01 (−0.15, 0.14)	−0.23 (−0.37, −0.09)	−0.16 (−0.30, −0.01)	−0.02 (−0.16, 0.13)	−0.06 (−0.21, 0.08)	−0.13 (−0.27, 0.02)	−0.13 (−0.27, 0.01)	−0.27 (−0.12, −0.41)
Born in Australia	No	**3.15 (0.53)**	2.94 (0.55)	3.03 (0.47)	2.98 (0.56)	2.78 (0.58)	**3.83 (0.77)**	**3.71 (0.75)**	**3.53 (0.84)**	**3.69 (0.84)**
		n = 262	n = 262	n = 260	n = 261	n = 260	n = 259	n = 260	n = 259	n = 260
	Yes	**3.25 (0.54)**	2.99 (0.54)	3.02 (0.51)	3.06 (0.55)	2.78 (0.52)	**4.04 (0.64)**	**3.88 (0.63)**	**3.71 (0.69)**	**3.94 (0.68)**
		n = 541	n = 540	n = 541	n = 541	n = 538	n = 533	n = 535	n = 533	n = 534
Effect size COB (95 %, CI)		−0.18 (−0.33, −0.03)	−0.10 (−0.25, 0.05)	0.02 (−0.13, 0.17)	−0.13 (−0.28, 0.01)	−0.01 (−0.16, 0.14)	−0.32 (−0.46, −0.17)	−0.26 (−0.41, −0.11)	−0.24 (−0.39, −0.09)	−0.33 (−0.48, −0.18)
English spoken at home	No	**3.04 (0.49)**	**2.81 (0.62)**	2.99 (0.49)	**2.87 (0.55)**	2.79 (0.60)	**3.37 (0.92)**	**3.39 (0.88)**	**3.18 (0.93)**	**3.16 (0.93)**
		n = 73	n = 73	n = 73	n = 73	n = 73	n = 72	n = 73	n = 73	n = 73
	Yes	**3.24 (0.53)**	**3.00 (0.53)**	3.03 (0.49)	**3.06 (0.55)**	2.79 (0.53)	**4.03 (0.63)**	**3.87 (0.63)**	**3.70 (0.71)**	**3.93 (0.69)**
		n = 721	n = 720	n = 720	n = 720	n = 717	n = 715	n = 718	n = 715	n = 717
Effect size English (95 %, CI)		−0.37 (−0.61, −0.12)	−0.35 (−0.59, −0.11)	−0.10 (−0.34, 0.14)	−0.33 (−0.57, −0.09)	0.01 (−0.23, 0.25)	−1.00 (−1.24, −0.75)	−0.72 (−0.97, −0.48)	−0.71 (−0.96, −0.47)	−1.09 (−1.33, −0.84)
Number of chronic conditions	<4	3.21 (0.53)	**3.00 (0.54)**	3.03 (0.49)	**3.06 (0.54)**	2.78 (0.55)	4.00 (0.69)	**3.86 (0.67)**	3.68 (0.73)	3.87 (0.76)
		n = 536	n = 535	n = 535	n = 535	n = 532	n = 528	n = 530	n = 527	n = 530
	≥4	3.22 (0.56)	**2.91 (0.55)**	3.00 (0.49)	**2.95 (0.58)**	2.78 (0.51)	3.90 (0.68)	**3.73 (0.65)**	3.58 (0.76)	3.82 (0.70)
		n = 252	n = 252	n = 252	n = 252	n = 251	n = 249	n = 250	n = 250	n = 249
Effect size condition (95 % CI)		−0.03 (−0.18, 0.12)	0.18 (0.03, 0.33)	0.06 (−0.09, 0.21)	0.21 (0.06, 0.36)	−0.00 (−0.15, 0.15)	0.14 (−0.01, 0.29)	0.20 (0.05, 0.35)	0.13 (−0.02, 0.28)	0.08 (−0.07, 0.23)
Lives alone	No	3.21 (0.54)	2.96 (0.53)	3.00 (0.48)	**3.09 (0.52)**	2.79 (0.53)	3.97 (0.69)	3.82 (0.68)	3.66 (0.72)	3.86 (0.73)
		n = 440	n = 440	n = 439	n = 439	n = 437	n = 437	n = 439	n = 438	n = 438
	Yes	3.22 (0.54)	3.00 (0.55)	3.06 (0.50)	**2.96 (0.59)**	2.77 (0.54)	3.98 (0.68)	3.83 (0.67)	3.65 (0.78)	3.86 (0.74)
		n = 336	n = 335	n = 335	n = 336	n = 334	n = 333	n = 335	n = 332	n = 335
Effect size lives alone (95 % CI)		−0.02 (−0.16, 0.13)	−0.07 (−0.21, 0.07)	−0.13 (−0.27, 0.01)	0.25 (0.11, 0.39)	0.05 (−0.09, 0.19)	−0.03 (−0.17, 0.12)	−0.02 (−0.16, 0.12)	0.02 (−0.13, 0.16)	−0.00 (−0.15, 0.14)
Lives in rural area	No	3.21 (0.51)	2.97 (0.53)	3.02 (0.50)	3.04 (0.55)	2.78 (0.53)	3.96 (0.70)	3.83 (0.67)	3.63 (0.76)	3.84 (0.76)
		n = 642	n = 641	n = 640	n = 641	n = 637	n = 637	n = 634	n = 632	n = 633
	Yes	3.22 (0.62)	3.01 (0.58)	3.04 (0.49)	3.01 (0.56)	2.81 (0.55)	4.00 (0.65)	3.81 (0.67)	3.74 (0.69)	3.91 (0.69)
		n = 169	n = 169	n = 169	n = 169	n = 169	n = 167	n = 167	n = 166	n = 167
Effect size or rural (95 % CI)		−0.03 (−0.20, 0.14)	−0.07 (−0.24, 0.10)	−0.05 (−0.22, 0.12)	0.04 (−0.13, 0.21)	−0.07 (−0.24, 0.10)	−0.05 (−0.22, 0.12)	0.02 (−0.15, 0.19)	−0.15 (−0.32, 0.02)	−0.09 (−0.26, 0.08)

Results in bold have p-value <0.05 for difference in means (tested using robust ANOVA); Effect size (ES) calculated using Cohen’s d for standardised difference in means. Interpretation of ES: “small” ES >0.20-0.50 SD, “medium” ES approximately 0.50-0.80 SD, and “large” ES >0.80 SD

The largest effect size for difference in means were seen between English and non-English speaking participants. These differences were observed across all scales of the HLQ with the exception of ‘Actively managing health’ and ‘Appraisal of health information’. Effect sizes [95 % CI] ranged from particularly large (‘Understanding health information well enough to know what to do’ ES = −1.09 [−1.33 to −0.84]; ‘Ability to actively engage with healthcare providers’ ES = −1.00 [−1.24, −0.75]) to medium (‘Social support for health’ ES = −0.33 [−0.57, −0.09]).

Similar patterns of lower health literacy indicators, mainly with medium effect sizes, were seen according to country of birth. Participants born overseas were more likely than Australian born participants to have lower scores in ‘Feeling understood and supported by healthcare providers’ (ES −0.18 [−0.33, −0.03]), ‘Ability to actively engage with healthcare providers’ (ES −0.32 [−0.46, −0.17]), ‘Ability to navigate the healthcare system’ (ES −0.26 [−0.41, −0.11]), ‘Ability to find good health information’ (ES −0.24 [−0.39, −0.09]), and ‘Understanding health information well enough to know what to do’ (ES −0.33 [−0.48, −0.18]).

Small to medium effect sizes were seen for differences in three HLQ scales according to educational level. Participants who had completed secondary education had higher scores than their less educated counterparts in ‘Appraisal of health information’ (ES 0.25 [0.11, 0.39]), ‘Ability to find good health information’ (ES 0.25 [0.11, 0.39]), and ‘Understanding health information well enough to know what to do’ (ES 0.28 [0.14, 0.41]). Compared with those with private health insurance, not having insurance was also associated with small to medium differences in ‘Actively managing health’ (ES −0.23 [−0.37, −0.09]), ‘Social support for health’ (ES −0.16 [−0.30, −0.01]), and ‘Understanding health information well enough to know what to do (ES −0.27 [−0.12, −0.41]).

The number of chronic health conditions reported by participants was associated with small effect sizes in three scales. Differences between participants reporting fewer than four conditions compared to those with four or more reported higher ‘Having sufficient information to manage health’ (ES 0.18 [0.03, 0.33]), ‘Social support for health’ (ES 0.21 [0.06, 0.36]), and ‘Navigating the healthcare system’ (ES 0.20 [0.05, 0.35]).

Several demographic groups were similar in their HLQ responses (i.e., effect sizes for differences in means were smaller across fewer HLQ scales). Compared to males, females had lower scores in the scales ‘Social support for health’ and ‘Ability to engage with healthcare providers’, although effect sizes were very small. Participants aged over 65 years were more likely to report ‘Actively managing health’ and having ‘Social support for health’ compared with younger participants. Living alone was associated with lower scores in ‘Social support for health’. No differences in HLQ scores were seen for participants living in rural compared to urban areas.

## Discussion

This study demonstrates small to very large differences in health literacy within demographic sub-groups of typical adult users of services in Victoria. Particular groups with the largest health literacy differentials compared to their counterparts are those born overseas, those not speaking English at home, and those with low education. Differences were also observed according to sex, age group, private health insurance status, number of chronic conditions, and living arrangements.

### Lower health literacy among vulnerable demographic groups

Certain demographic groups are well known to be vulnerable in terms of health inequalities [41–44]. In our study, groups with lower health literacy include those born overseas or who speak languages other than English at home, those with lower education or no private health insurance, those with multiple chronic conditions, and women. These groups reported difficulties actively engaging with healthcare providers, navigating the healthcare system, finding or understanding health information and in having adequate social support for health. Such population sub-groups are already known to be disadvantaged in terms of health outcomes and access to health services, and it may be that these disparities have been caused, at least in part, by the identified elements of low health literacy [45]. Our findings identify the particular health literacy domains that may contribute to inequities and highlight that in settings where organisations provide universal or ‘one size fits all’ healthcare services or programs, important subgroups may be strongly disadvantaged.

### Health literacy difficulties among migrant participants

We found very large differences in health literacy scores for participants born in countries other than Australia and those for whom English was not their first language. Both these groups scored lower than their counterparts on scales focused on relationships with healthcare providers, navigation of the healthcare system, and finding or understanding health information. It is important to note that these differences are very large, with some effect sizes being as high as 0.70 to 1.00. In health-related areas, effect sizes of such magnitude are uncommon [46]. Many previous studies indicate that people from culturally and linguistically diverse (CALD) communities are less likely to access necessary services or understand issues related to their health and are at greater risk of mismanaging their medications [47]. This may particularly be the case for older adults from culturally diverse backgrounds [48]. People from CALD communities are often unfamiliar with the role of the health system and healthcare providers, may be exposed to culturally inappropriate services, or may have language difficulties [48]. Furthermore, the well-documented phenomenon of the rapid decline in the health of initially healthy migrants [49] may in part be explained by the health literacy dimensions we have identified. While this decline in health is thought to be partly due to the statistical phenomenon of regression to the mean [50], evidence suggests other factors may also play a role. These include environmental, cultural or behavioural factors [51] and health literacy [52]. Our findings provide novel insights into mechanisms that may be operating to create disparities for CALD communities. The range of health literacy limitations identified indicate that supporting CALD clients to overcome health literacy-related barriers to access will require broad approaches at individual, organisational and policy levels. Focusing on one aspect only (such as provision of translated health information), is unlikely to yield positive outcomes given the complexity of the health literacy difficulties experienced by CALD clients and communities.

### Relationships between health literacy and socioeconomic status

Specific indicators of socioeconomic status in our study were level of education and private health insurance status. We found that those who had completed secondary education scored more highly in the three domains relating to finding, understanding and appraising health information compared with their less well educated counterparts. Educational attainment has long been associated with health literacy, as measured using earlier tools [44]. This is not surprising given that the majority of these earlier tools focus on numeracy and reading comprehension only. Our findings suggest that whilst less educated clients experience limitations in several health literacy domains, their skills are equal to more highly educated clients in other domains. An important question for further study is whether (and for whom, and under which circumstances) these strengths might act to compensate for observed limitations.

Private health insurance is also regarded as a proxy indicator for socioeconomic status [53]. Few studies have explored the relationship between private health insurance and health literacy; none of these from Australia [54]. We found that participants with private health insurance scored more highly than those without insurance in the domains of actively managing health, social support, and understanding health information. Similar to our findings, a qualitative study from North America found that people without private health insurance were less likely to engage in active self-management compared with those with private insurance [54]. Our results may reflect the type of skills required to obtain health insurance, and the type of clients that proactively seek insurance, or are more likely to be attracted by the benefits of health insurance. Reducing the complexity of information relating to health insurance plans and their benefits may address the disparities observed in the current study.

### Health literacy in specific groups of people

Clients with four or more chronic conditions also reported more difficulties in navigating the healthcare system, having sufficient information for health, and having less social support for health. Studies identify that having attentive, ‘quality’ social support (as perceived by patients with chronic disease) is associated with better health outcomes [55] and improved self-management [56–58]. Providing education and support for family members and carers may be a useful strategy for health services to consider, as systematic reviews suggest that information needs of carers of people with chronic disease are high [59, 60]. The relatively low scores seen for navigating the healthcare system and having enough information to manage health may relate to being overwhelmed by the complexity of information and the number of service providers involved in their care. Potential strategies to address this include the use of patient navigators to support patients through the system [61], or the use of online methods of information delivery [62].

### Comparison with other studies

Our results are consistent with other studies using the HLQ. A recent Danish study integrated two HLQ scales into a population-based health survey (‘Understanding health information well enough to know what to do’, and ‘Ability to actively engage with healthcare providers’) [63]. Similar to our results, this study found that being born in another country, not speaking Danish, and having lower education were associated with lower scores in both scales. Our results are also consistent with earlier health literacy studies, although it is important to note that these studies used health literacy measurement tools focusing on reading comprehension and numeracy skills only. Older age has also been shown in several studies to be associated with lower health literacy [7, 11, 64] (although this depends on the health literacy measurement tool used) [11], as is lower education [7, 11, 65] and not having private health insurance [64]. Findings are less consistent for the relationship between gender and health literacy, with some studies demonstrating no association [11, 65], and others demonstrating that compared with men, women may have higher [64] or lower health literacy [65].

### Strengths and limitations

This the first study to describe the health literacy of adults using a measure of health literacy that provides robust multi-dimensional profiles. Data from the HLQ has not only identified the specific areas in which particular groups of clients experience sub-optimal health literacy, but has also provided indications of which issues need to be addressed to improve health and equity outcomes. The large sample allows for sub-group analysis, enabling the identification of key intervention targets for organisations seeking to address health literacy needs within their community. Importantly, the HLQ provides largely unbiased estimates of mean differences in composite scores between groups [36]. This is particularly important, because individuals from different community sub-groups may interpret the meaning and content of questionnaire items differently. When bias such as this is present in data, group differences may be contaminated by cultural or linguistic factors. We have found that the HLQ scales provide equivalent measurement across groups, with only minor bias in scales 7 to 9 [36]. In practice, this means that small over-estimates of health literacy may be occurring in people with lower levels of education from non-English backgrounds.

While these results demonstrate that health literacy scores differ between demographic groups, it is important to acknowledge that we do not yet know what a clinically meaningful difference in scores could be, other than the convention that a 0.5 ES is often regarded as ‘significant’ [66–68]. Further, even when HLQ scale scores are low for a particular group we cannot assume that this represents a need. This is because some individuals may adequately compensate for a limitation in one health literacy domain through other strengths, including in other health literacy domains [69].

Although the sample is not representative of the Victorian population as a whole, the selected organisations are typical of those providing care to older people and those with chronic disease. Furthermore, the organisations were encouraged to collect HLQ data from a representative sample of their target group, with substantial efforts to collect data from the ‘harder to reach’ clients. As this was a pragmatic study in typical healthcare settings, data on clients who declined to participate were not readily available and so we were unable to compare differences between participants and non-participants. However, as seen in other studies [70–72], it is probable that clients with lower health literacy were less well represented, indicating our findings may be an underestimation of the problem. In addition, despite the fact that the HLQ was translated into many of the common languages spoken in Victoria, the number of non-English speaking participants was lower than in the general population (90.8 % of our sample spoke English at home, compared to 72.4 % in Victoria in 2011) [33]. Further, interpreters were not readily available to orally administer the HLQ to illiterate non-English speakers, and as such these clients were less likely to have been included in the sample. This again may have led to an underestimation of the challenges experienced by this group. Further, it is probable that the most engaged and skilled migrants chose to participate, again potentially contributing to underestimation of the differences across some sub-groups. Data were not collected about the year of migration to Australia, and it may be that more recent arrivals experience greater difficulty than those who have lived in Australia for some time.

Approximately 37 % of the sample were assisted to answer the questionnaire for reasons including vision or physical impairment, stroke or illiteracy. Given that data collection was undertaken by staff from each site, or in some cases, questionnaires were mailed to participants, reasons for assistance were not systematically documented. It is possible that being provided with assistance may have influenced respondents’ answers, particularly for items related to feeling understood and supported by healthcare providers. However, no statistically significant differences were seen between mean scores for this particular scale in those who were and were not assisted to complete the questionnaire. Further, the efforts taken to reach into this harder-to-reach group ensured that the people in the study were more typical of people attending services on a daily basis.

## Implications

Our findings provide insight into key areas in which people can be supported to access, understand and use health information, and have implications for clinicians and organisations seeking to improve health outcomes and reduce health inequalities for clients with chronic disease. Findings are particularly relevant for healthcare organisations aiming to improve access for client groups that are less engaged with services. Key service access points include when clients first approach a service, receive information, decide to participate in proactive care such as preventive or self–management activities, and when they engage with healthcare providers [32]. The identification of areas of difficulty for particular demographic groups make these findings highly relevant for organisations or practitioners seeking to intervene at any of the above access points.

## Conclusion

This study demonstrates the utility of the HLQ to identify health literacy strengths and difficulties of an adult population, most of whom had an existing health condition. This is in keeping with modern, broad definitions of health literacy that encompass the range of personal skills and social resources required to engage effectively with health information and services. The results demonstrate that important sub-groups within the population may be at greater risk of having fewer health literacy skills and resources than others. Our findings provide some practical guidance to healthcare services and policy makers and underline the potentially important role of health literacy in the development of equitable interventions to improve health outcomes.

## Abbreviations

HLQ: Health Literacy Questionnaire
ES: Effect size
CALD: culturally and linguistically diverse
